# A glucose-sensing mechanism with glucose transporter 1 and pyruvate kinase in the area postrema regulates hepatic glucose production in rats

**DOI:** 10.1016/j.jbc.2023.104633

**Published:** 2023-03-23

**Authors:** Rosa J.W. Li, Jennifer F.M. Chiu, Kyla Bruce, Song-Yang Zhang, Daniel R. Barros, Jessica T.Y. Yue, Tony K.T. Lam

**Affiliations:** 1 Department of Physiology, University of Toronto, Toronto, Canada; 2 Toronto General Hospital Research Institute, UHN, Toronto, Canada; 3 Institute of Medical Science, University of Toronto, Toronto, Canada; 4 Department of Physiology, University of Alberta, Edmonton, Canada; 5 Banting and Best Diabetes Centre, University of Toronto, Toronto, Canada

**Keywords:** area postrema, nucleus tractus solitarius, glucose transporter 1, pyruvate metabolism, glucose production

## Abstract

The area postrema (AP) of the brain is exposed to circulating metabolites and hormones. However, whether AP detects glucose changes to exert biological responses remains unknown. Its neighboring nuclei, the nucleus tractus solitarius (NTS), responds to acute glucose infusion by inhibiting hepatic glucose production, but the mechanism also remains elusive. Herein, we characterized AP and NTS glucose-sensing mechanisms. Infusion of glucose into the AP, like the NTS, of chow rats suppressed glucose production during the pancreatic (basal insulin)-euglycemic clamps. Glucose transporter 1 or pyruvate kinase lentiviral-mediated knockdown in the AP negated AP glucose infusion to lower glucose production, while the glucoregulatory effect of NTS glucose infusion was also negated by knocking down glucose transporter 1 or pyruvate kinase in the NTS. Furthermore, we determined that high-fat (HF) feeding disrupts glucose infusion to lower glucose production in association with a modest reduction in the expression of glucose transporter 1, but not pyruvate kinase, in the AP and NTS. However, pyruvate dehydrogenase activator dichloroacetate infusion into the AP or NTS that enhanced downstream pyruvate metabolism and recapitulated the glucoregulatory effect of glucose in chow rats still failed to lower glucose production in HF rats. We discovered that a glucose transporter 1– and pyruvate kinase–dependent glucose-sensing mechanism in the AP (as well as the NTS) lowers glucose production in chow rats and that HF disrupts the glucose-sensing mechanism that is downstream of pyruvate metabolism in the AP and NTS. These findings highlight the role of AP and NTS in mediating glucose to regulate hepatic glucose production.

The area postrema (AP) is a circumventricular organ located in the hindbrain that bypasses the blood-brain barrier and exposes to nutrients and hormones. AP is situated next to the nucleus tractus solitarius (NTS) and the dorsal motor nucleus of the vagus nerve that compose of the dorsal vagal complex. Hormones such as glucagon-like peptide 1, amylin, and growth differentiation factor-15 activate receptors expressed in the AP to regulate food intake (1, 2, 3, 4).

Feeding induces cFOS expression in the AP as well (5). In fact, AP contains glucose-sensitive neurons that respond to glucose changes and alter firing rate (6), and AP lesion prevents glucoprivic signals to stimulate feeding (7). In parallel, glucoprivic signals in the NTS increase food intake (8) but also activate counterregulatory responses to elevate plasma glucose levels (9). NTS glucose infusion that increases local glucose levels to a similar extent as seen with systemic hyperglycemia suppresses glucose production in rats as well (10). Similarly, the hypothalamus detects changes in glucose and fatty acid levels to not only regulate food intake but also hepatic glucose production (11, 12, 13, 14, 15). However, whether the AP regulates glucose homeostasis remains unknown.

Hypothalamic glucose infusion suppresses glucose production *via* glial glucose transporter 1 (GLUT1)-mediated glucose uptake (16). Blunted hypothalamic glucose uptake impairs hypothalamic glucose-sensing, associated with decreased brain or hypothalamic GLUT1 levels in rodent models of short-term high-feeding and uncontrolled diabetes, respectively (16, 17). Despite these findings and the wide expression of GLUT1 in the brain endothelial cells and parenchymal glial cells (18), limited studies have examined its involvement in central nutrient-sensing and metabolic regulation. Upon cellular uptake of glucose, subsequent metabolism to lactate -> pyruvate -> acetyl-CoA is sufficient and necessary for hypothalamic glucose-sensing to inhibit glucose production in chow male rats (11). In contrast, neither lactate infusion nor the disruption of lactate metabolism in the presence of glucose in the NTS alter glucose production (10). However, whether GLUT1 or pyruvate metabolism is required for NTS and/or potentially AP glucose-sensing mechanism to regulate glucose homeostasis remains to be investigated. In addition, 3 days of high-fat (HF) feeding in male rats develop hyperphagia and brain insulin resistance as well as failing to response to hypothalamic glucose and oleic acid infusion to lower glucose production prior to the onset of obesity (14, 19, 20, 21). It remains unknown as well whether short-term HF feeding impairs sensory mechanism in the AP and/or NTS to disrupt glucose production regulation.

In this study, we set out to examine in rats whether AP, like NTS, detects an acute rise of glucose to regulate glucose metabolism, assess the necessary roles of GLUT1 and pyruvate metabolism in AP and NTS for glucose homeostasis, and dissect the underlying mechanism of short-term HF-induced potential defect(s) in AP and NTS *in vivo*.

## Results

### Lentiviral-mediated knockdown of GLUT1 in AP negates glucose infusion to lower glucose production

**Figure 1:**
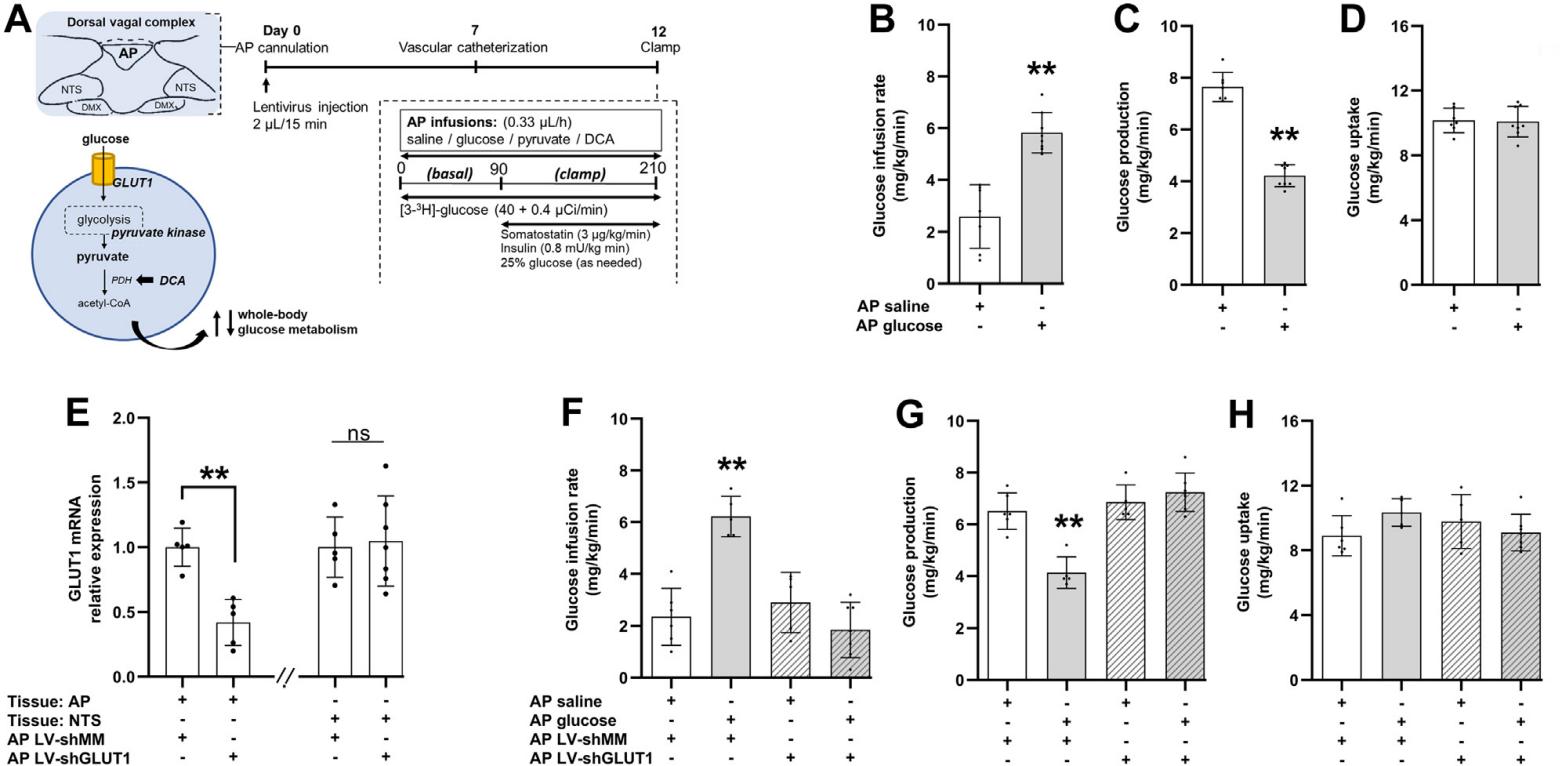
**Lentiviral-mediated knockdown of GLUT1 in AP negates glucose infusion to lower glucose production.***A*, schematic representation of working hypothesis: AP glucose infusion modulates whole-body glucose metabolism *via* GLUT1 and pyruvate metabolism-dependent mechanisms. Experimental protocol of pancreatic (basal insulin)-euglycemic clamp experiments with rats receiving AP infusions. *B*, glucose infusion rate, (*C*) glucose production, and (*D*) glucose uptake of rats receiving AP saline (n = 7) or glucose (n = 8) during clamps (please refer to the Experimental procedures section for details on calculations). *E*, relative mRNA expression of GLUT1 in AP tissue of rats receiving AP-specific lentiviral infection of scrambled mismatch sequence (LV-shMM) (n = 5) or AP shRNA of GLUT1 (LV-shGLUT1) (n = 5) and nucleus tractus solitarius (NTS) tissue of rats receiving AP LV-shMM (n = 5) or AP LV-shGLUT1 (n = 7) normalized to 18s rRNA. *F*, glucose infusion rate, (*G*) glucose production, and (*H*) glucose uptake of rats during clamps receiving AP infusion of saline with AP infection of LV-shMM (n = 6), AP glucose with AP LV-shMM (n = 5), AP saline with AP LV-shGLUT1 (n = 5), or AP glucose with AP LV-shGLUT1 (n = 7). ∗∗*p* < 0.01 *versus* other groups determined by unpaired *t* test or one way ANOVA followed by Tukey’s post hoc test. Data are shown as mean ± S.D. AP, area postrema; DCA, dichloroacetate; DMX, dorsal nucleus of motor nerve; GLUT1, glucose transporter-1; PDH, pyruvate dehydrogenase.

To examine whether the AP detects glucose to regulate systemic glucose metabolism, we infused glucose into AP of conscious, unrestrained male rats and performed pancreatic (basal insulin)-euglycemic clamps to assess for changes in glucose production and uptake (Fig. 1*A*). We first verified that the brain infusion selectively targeted the AP by infusing bromophenol blue dye *via* the cannula each time after experiment (Fig. S1*A*). Rats that did not have dye localized within the AP were excluded.

The dosage of glucose (2 mM) infused into the AP is documented to activate NTS and hypothalamic glucose sensing to a comparable extent as systemic hyperglycemia (10, 22). Herein, we found that glucose (2 mM) *versus* saline infusion into the AP increased the exogenous glucose infusion rate required to maintain euglycemia during the pancreatic (basal-insulin) clamps (Fig. 1*B* and Table S1), indicating that AP glucose infused at this dose not only did not leak into the circulation but led to a drop in plasma glucose levels. This glucose-lowering effect by AP glucose infusion was due to a suppression of glucose production (Fig. 1*C*) but not an increase in glucose uptake (Fig. 1*D*) nor changes in basal glucose production (Fig. S1*D*), while the rats had comparable food intake and bodyweight prior to the studies (Fig. S1, *B* and *C*). C-peptide levels were comparable among groups during the basal period and were significantly reduced during the clamps by somatostatin, altogether indicating that endogenous insulin secretion is potently suppressed in our model (Table S2). Taken together, this is the first evidence to show that the AP is sensitive to an acute rise in glucose levels to regulate systemic glucose metabolism.

Next, we employed a genetic tool by injecting lentiviruses carrying shRNA of GLUT1 (LV-shGLUT1) or scrambled mismatch sequence (LV-shMM) as control into the AP to not only investigate the necessary role of GLUT1 in AP glucose sensing but also alternatively evaluate the spread of the infusion. We found that AP LV-shGLUT1 injection successfully knocked down the level of GLUT1 in AP by ∼50% compared to LV-shMM–injected rats, but not in the neighboring nuclei NTS (Fig. 1*E*). In rats that received LV-shMM, AP glucose *versus* saline infusion increased exogenous glucose infusion rate during the clamps by suppressing glucose production but not an increase in glucose uptake (Fig. 1, *F*–*H*). These effects were comparable to nonviral-injected rats (Fig. 1, *C* and *D*), indicating that viral infection *per se* in the AP in the current experimental conditions did not alter AP glucose-sensing mechanism. Importantly, AP glucose infusion failed to increase glucose infusion rate and lower glucose production in rats injected with LV-shGLUT1 (Fig. 1, *F*–*H*), while food intake and bodyweight were comparable among groups prior to the studies (Fig. S1, *E* and *F*). Thus, GLUT1 in the AP is required for glucose infusion in the AP to lower glucose production.

### Lentiviral-mediated knockdown of GLUT1 in NTS negates glucose infusion to lower glucose production

**Figure 2:**
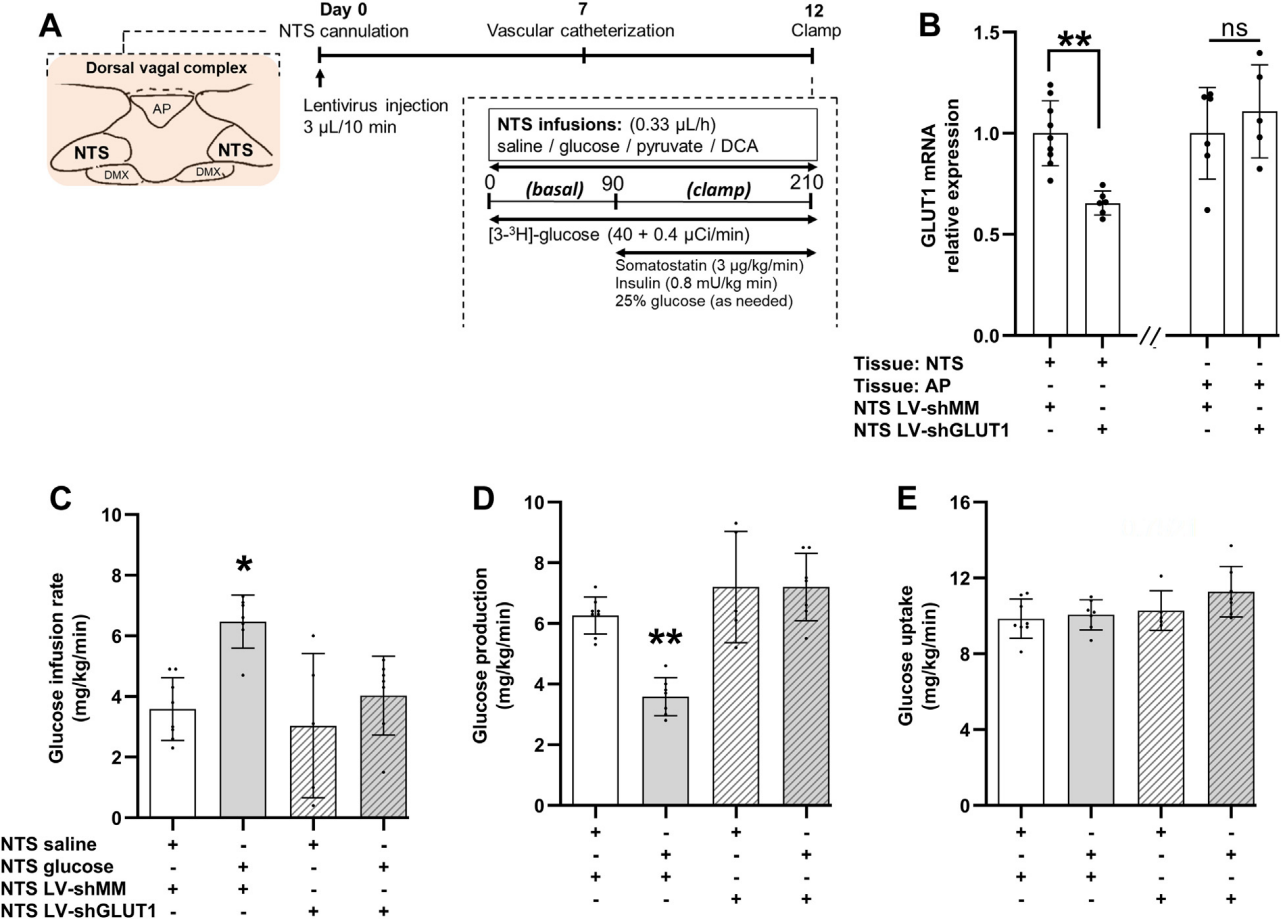
**Lentiviral-mediated knockdown of GLUT1 in NTS negates glucose infusion to lower glucose production.***A*, experimental protocol of pancreatic (basal insulin)-euglycemic clamp experiments with rats receiving NTS infusions. *B*, relative mRNA expression of GLUT1 in NTS tissue receiving NTS lentiviral infection of LV-shMM (n = 9) or NTS LV-GLUT1 (n = 11) and AP tissues receiving NTS LV-shMM (n = 6) or NTS LV-shGLUT1 (n = 9) normalized to 18s rRNA. *C*, glucose infusion rate, (*D*) glucose production, and (*E*) glucose uptake during the clamps of rats receiving NTS saline with NTS LV-shMM infections (n = 8), NTS glucose with NTS LV-shMM (n = 7), NTS saline with NTS LV-shGLUT1 (n = 5), or NTS glucose with NTS LV-shGLUT1 (n= 7). ∗*p* < 0.05, ∗∗*p* < 0.01 *versus* other groups determined by unpaired *t* test or one way ANOVA followed by Tukey’s post hoc test. Data are shown as mean ± S.D. AP, area postrema; GLUT1, glucose transporter-1; NTS, nucleus tractus solitarius.

To assess the role of GLUT1 in glucose sensing in the NTS, we injected LV-shGLUT1 *versus* LV-shMM into the NTS of chow rats and performed pancreatic clamps with NTS saline or glucose infusions (Fig. 2*A*). We ensured that NTS glucose infusion is specific to the NTS as demonstrated by the dye image provided (Fig. S2*A*). Alternatively, GLUT1 mRNA expression was knocked down by ∼40% in the NTS of chow male rats injected with LV-shGLUT1 *versus* LV-shMM into the NTS, while GLUT1 mRNA expression in the adjacent nuclei AP was unaffected (Fig. 2*B*).

In the presence of LV-shMM injection, NTS-targeted glucose *versus* saline infusion increased glucose infusion rate during the clamps by suppressing glucose production (Fig. 2, *C*–*E* and Table S1), and these glucoregulatory effect of NTS glucose infusion was comparable in nonviral-injected rats (10). NTS glucose infusion failed to increase glucose infusion rate and lower glucose production in LV-shGLUT1–injected rats with GLUT1 knocked down in the NTS (Fig. 2, *C* and *D* and Table S1). Glucose uptake as well as food intake and bodyweight prior to the studies were comparable among groups (Figs. 2*E* and S2, *B* and *C*). Interestingly, basal glucose production (60–90 min, Fig. S2*D*) prior to the clamps was modestly elevated in rats that received LV-shGLUT1 with NTS glucose *versus* saline infusion, an effect that was not detected in AP GLUT1 knocked down rats (Fig. S1*G*). Nonetheless, we discovered that like in the AP, GLUT1 in the NTS is required for glucose infusion to lower glucose production.

### Pyruvate formation is necessary for AP and NTS glucose sensing to regulate glucose metabolism

**Figure 3:**
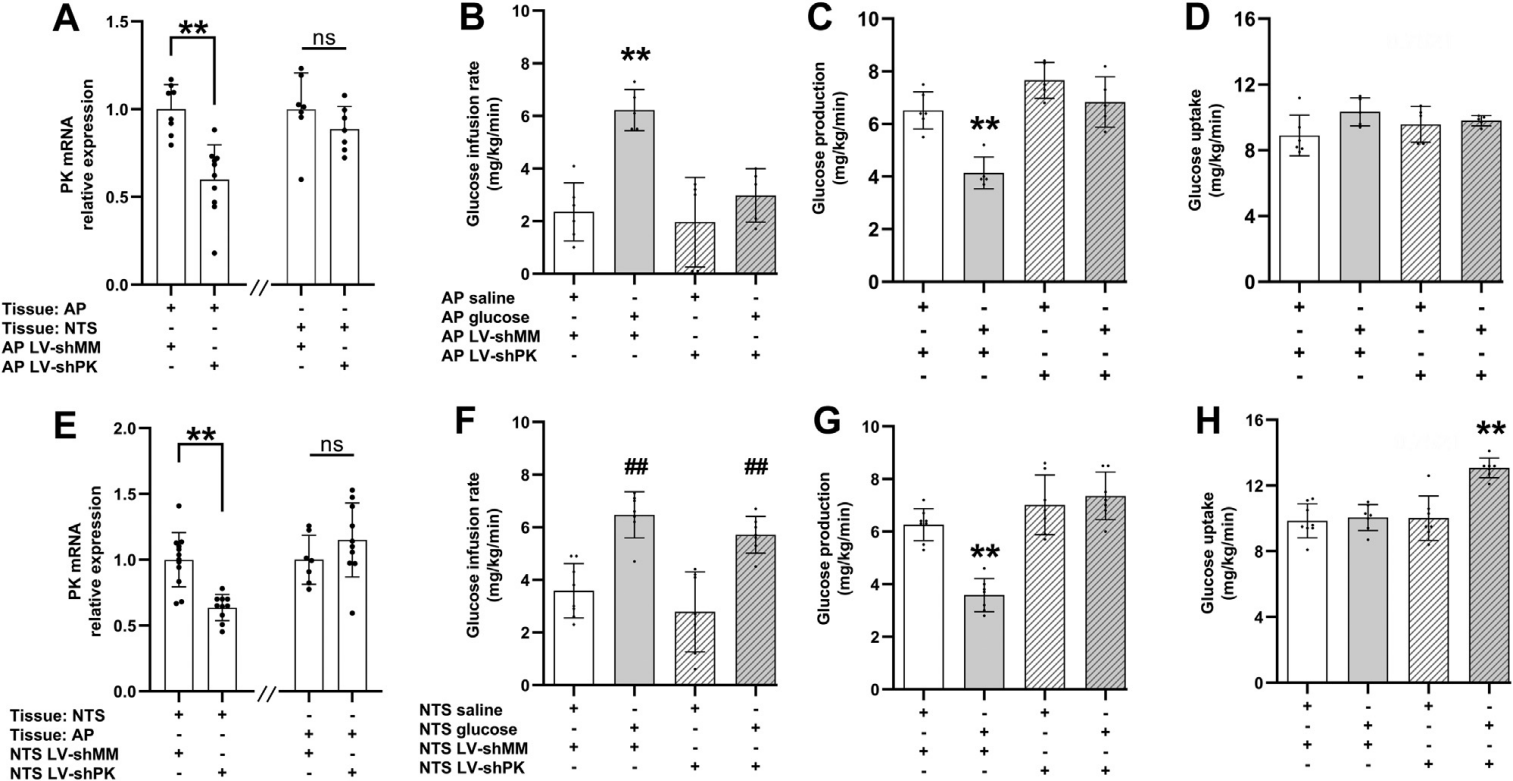
**Pyruvate kinase is required for AP and NTS glucose infusion to regulate glucose metabolism.***A*, relative mRNA expression of pyruvate kinase in AP tissues receiving AP LV-shMM (n = 8) or AP LV-shPK (n = 10) and NTS tissues receiving AP LV-shMM (n = 7) or AP LV-shPK (n = 7) normalized to 18s rRNA. *B*, glucose infusion rate, (*C*) glucose production, (*D*) glucose uptake during clamp steady-state of rats receiving AP saline with AP LV-shMM (n = 6), AP glucose with AP LV-shMM (n = 5), AP saline with AP lentiviral infection of shRNA of pyruvate kinase (LV-shPK) (n = 5), and AP glucose with AP LV-shPK (n = 5). *E*, relative mRNA expression of pyruvate kinase in NTS tissues receiving NTS LV-shMM (n = 12) or NTS LV-shPK (n = 12) and AP tissues receiving NTS LV-shMM (n = 7) or NTS LV-shPK (n = 12) normalized to 18s rRNA. *F*, glucose infusion rate, (*G*) glucose production, and (*H*) glucose uptake during the clamp steady-state of rats receiving NTS saline with NTS LV-shMM (n = 8), NTS glucose with NTS LV-shMM (n = 7), NTS saline with NTS LV-shMM (n = 7), and NTS glucose with NTS LV-shPK (n = 7). ∗∗*p* < 0.01 *versus* other groups, ##*p* < 0.01 *versus* NTS saline with NTS LV-shMM or NTS LV-shPK determined by unpaired *t* test or one way ANOVA followed by Tukey’s post hoc test. Data are shown as mean ± S.D. AP, area postrema; NTS, nucleus tractus solitarius.

To examine the necessary subsequent step(s) upon cellular glucose entry, we investigated whether glucose metabolism into pyruvate *via* pyruvate kinase is required for AP and NTS glucose sensing. We performed AP injection of lentivirus carrying shRNA of pyruvate kinase (LV-shPK) *versus* LV-shMM to knockdown pyruvate kinase and inhibit the formation of pyruvate in response to saline *versus* glucose infusion into the AP (Fig. 1*A*). We first confirmed that AP LV-shPK *versus* LV-shMM injection reduced pyruvate kinase expression by ∼40% in the AP but not in the NTS (Fig. 3*A*). We found that infusion of glucose *versus* saline into the AP failed to increase glucose infusion rate and lower glucose production during the clamps in rats injected with AP LV-shPK vs LV-shMM with comparable glucose uptake (Fig. 3, *B*–*D* and Table S1). Basal glucose production as well as food intake and bodyweight prior to the clamp studies were comparable among groups (Fig. S3). Taken together, pyruvate kinase–mediated pyruvate formation is necessary for AP glucose infusion to lower glucose production.

Next, we repeated the studies targeting the NTS. Tissue analysis first confirmed that pyruvate kinase was knocked down by ∼50% in the NTS but not the AP in NTS LV-shPK– *versus* LV-shMM–injected rats (Fig. 3*E*). Interestingly, we discovered that NTS glucose infusion was still able to increase glucose infusion rate during the pancreatic-euglycemic clamps in rats injected with NTS LV-shPK to a comparable extent as seen in rats injected with NTS LV-shMM (Fig. 3*F* and Table S1). However, NTS glucose infusion failed to lower glucose production in NTS LV-shPK- *versus* LV-shMM-injected rats *but* increased glucose uptake (Fig. 3, *G* and *H*), which led to the increase in glucose infusion rate (Fig. 3*F*). Of note, NTS glucose infusion was found *not to* increase glucose uptake in nonviral- or LV-shMM–injected rats (Figs. 2*E* and 3*H*), while basal glucose production was also elevated in rats with NTS LV-shPK *versus* LV-shMM in glucose but not in saline-infused rats (Fig. S3*F*). Food intake and bodyweight prior to the clamp studies were comparable among groups (Fig. S3, *D* and *E*). Taken together, pyruvate formation is required for AP and NTS glucose infusion to lower glucose production.

### Pyruvate metabolism in the AP and NTS is sufficient to regulate glucose metabolism

**Figure 4:**
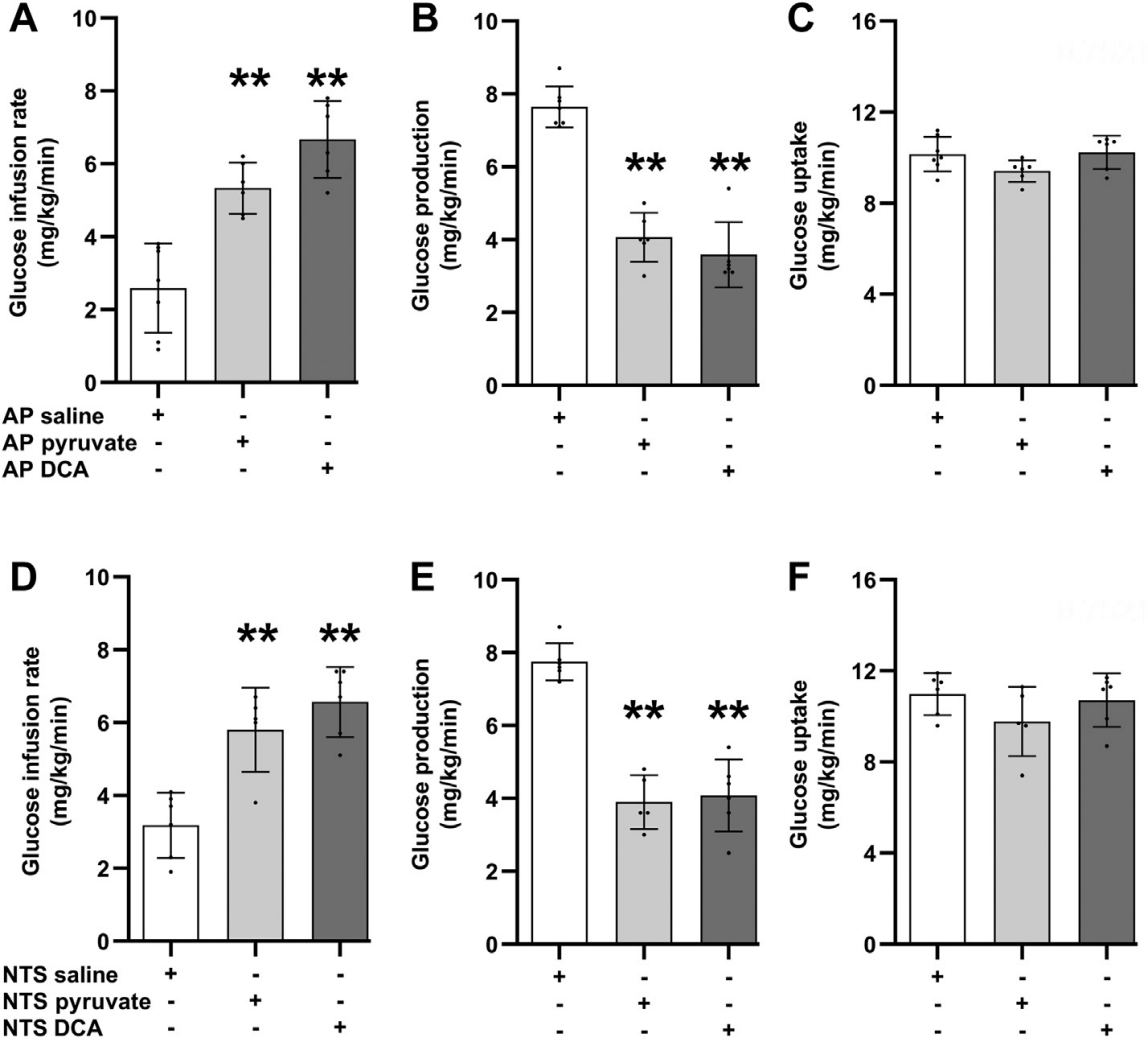
**Pyruvate metabolism in the AP and NTS is sufficient to regulate glucose metabolism.***A*, glucose infusion rate, (*B*) glucose production, and (*C*) glucose uptake of rats during clamp steady-state receiving AP saline (n = 7), AP pyruvate (n = 6), or AP DCA (n = 6). *D*, glucose infusion rate, (*E*) glucose production, and (*F*) glucose uptake of rats during clamp steady-state receiving NTS saline (n = 6), NTS pyruvate (n = 5), or NTS DCA (n = 6). ∗∗*p* < 0.01 *versus* respective saline group determined one way ANOVA followed by Tukey’s post hoc test. Data are shown as mean ± S.D. AP, area postrema; NTS, nucleus tractus solitarius.

To examine whether downstream pyruvate metabolism in the AP and NTS regulates glucose metabolism, we first infused pyruvate (5 mM) in the AP or NTS. Infusion of pyruvate *versus* saline into both AP and NTS was sufficient to recapitulate the effect of glucose by increasing glucose infusion rate and lowering glucose production during the euglycemic clamps. Meanwhile, no changes in glucose uptake or food intake and bodyweight prior to the studies were detected between the groups (Figs. 4, *A*–*F* and S4, *A*–*F*; Table S1).

Secondly, we infused the pyruvate dehydrogenase kinase inhibitor dichloroacetate to stimulate pyruvate conversion to acetyl-CoA in the AP or NTS. Like pyruvate infusion, both AP and NTS infusion of dichloroacetate increased glucose infusion rate and suppressed glucose production during the clamps, while glucose uptake and food intake and bodyweight prior to the studies remain comparable among the groups (Figs. 4, *A*–*F* and S4, *A*–*F*; Table S1). These results support the notion that increased pyruvate metabolism to acetyl-CoA, instead of elevated pyruvate levels *per se*, mimics glucose sensing in AP and NTS to suppress glucose production.

### Disruption of AP and NTS glucose-sensing mechanism by HF feeding

We next fed male rats that received AP or NTS surgeries with a lard oil–enriched HF diet for 3 days to induce hyperphagia and achieve an early-onset model of obesity, as bodyweight was not yet significantly elevated (Fig. S5, *A*–*D*). In this 3 days HF-fed rat model, studies have documented that the insulin signaling as well as glucose- and fatty acid–sensing mechanisms in the hypothalamus are impaired that led to a disruption in systemic glucose homeostasis (14, 19, 20).

**Figure 5:**
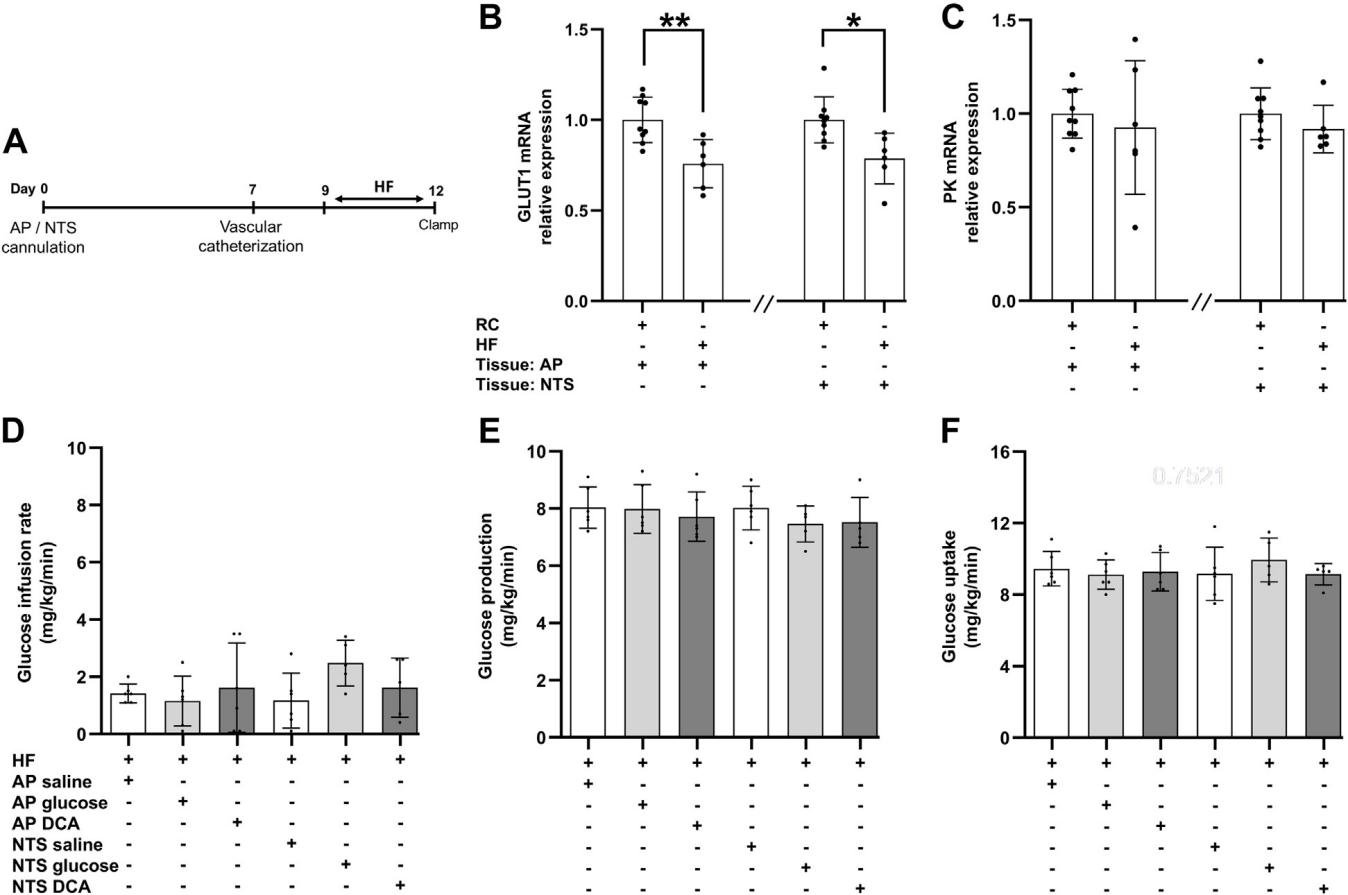
**Short-term high fat feeding downregulates GLUT1 expression and disrupts AP and NTS glucose sensing mechanism.***A*, experimental timeline for HF animals. Relative mRNA expression of (*B*) GLUT1 and (*C*) pyruvate kinase for rats fed with regular chow (RC) in AP (n = 13) or NTS tissues (n = 13) and HF in AP (n = 8) or NTS tissues (n = 10) normalized to 18s rRNA. ∗*p* < 0.05, ∗∗*p* < 0.01 as determined by unpaired *t* test. *D*, glucose infusion rate, (*E*) glucose production, and (*F*) glucose uptake of HF rats during clamp steady-state receiving AP saline (n = 6), AP glucose (n = 6), AP DCA (n = 6), NTS saline (n = 6), NTS glucose (n = 5), or NTS DCA (n = 5). Data are shown as mean ± S.D. AP, area postrema; GLUT1, glucose transporter-1; HF, high-fat; NTS, nucleus tractus solitarius.

Herein, we first discovered that 3 days HF *versus* chow feeding decreased GLUT1 expression in the AP and NTS by ∼20% but did not alter pyruvate kinase expression (Fig. 5, *B* and *C*). Next, we infused glucose (2 mM) into the AP of 3 days HF-fed rats and found that glucose failed to regulate systemic glucose metabolism (Fig. 5, *D*–*F*). We detected a lack of glucoregulatory effect when glucose was infused into the NTS of 3 days HF-fed rats as well (Fig. 5, *D*–*F*) as previously reported (10).

To evaluate whether the 20% decrease in GLUT1 expression in the AP and NTS was responsible for HF-induced disruption in AP and NTS glucose sensing, we activated the downstream pyruvate flux to acetyl-CoA by infusing dichloroacetate in the AP and NTS of 3 days HF-fed rats. Unexpectedly, dichloroacetate infusion into the AP and NTS still failed to regulate glucose metabolism (Fig. 5, *D*–*F*), particularly given that 3 days HF feeding did not alter pyruvate kinase expression in the AP and NTS. Our findings indicate that short-term HF feeding disrupts glucose sensing in the AP and NTS to lower glucose production by impairing local pathway(s) downstream of pyruvate metabolism.

## Discussion

In this study, we found that AP, like NTS (10), of male rats detects an acute rise in glucose levels to suppress glucose production. GLUT1 and pyruvate kinase–mediated formation of pyruvate in the AP and NTS are required for glucose sensing, while short-term HF feeding disrupts both AP and NTS glucose sensing in association with a modest reduction in GLUT1 expression but no changes in pyruvate kinase. However, direct enhancement of downstream pyruvate metabolism to acetyl-CoA *via* dichloroacetate infusion only lowered glucose production in chow but not in HF rats. It would be important to measure acetyl-CoA levels in the AP and NTS in future studies to investigate whether formation of acetyl-CoA is (i) necessary for glucose sensing to regulate hepatic glucose production and (ii) a potential site of impairment in HF rats, particularly given that HF feeding decreases acetyl-CoA levels in liver, muscle, and pancreatic tissues (23). Nonetheless, we propose glucose-sensing mechanistic defect(s) in the AP and NTS lie at the level or downstream of pyruvate flux to acetyl-CoA in response to HF feeding.

It is becoming apparent that the individual nucleus should be examined separately as they harbor distinct expression profiles and functions. For instance, acute leptin injection into the NTS suppresses feeding but fails to do so in the AP (24). GLP-1 receptor agonism differentially induces gene expression changes in the AP *versus* NTS (1), while the growth differentiation factor-15 receptor is much more highly expressed in the AP than NTS and suppresses food intake when infused into AP but not NTS (3). We hereby discovered that *both* AP and NTS detect glucose to regulate glucose production *via* GLUT1- and pyruvate kinase–dependent pathways.

While lactate metabolism is neither sufficient nor necessary for NTS glucose sensing (10), this is not the case for the hypothalamus (11, 19). Interestingly, basal glucose production was elevated in NTS glucose-infused rats but not in saline-infused rats when NTS GLUT1 or pyruvate kinase was knocked down, while basal glucose production was not altered in AP glucose-infused rats with GLUT1 or pyruvate kinase knocked down in the AP. Overall, although the role of lactate metabolism for AP and the necessary role of GLUT1 and pyruvate kinase for hypothalamic glucose sensing remain unclear, a difference in compensatory biochemical pathways could be engaged in the AP *versus* NTS *versus* hypothalamus that lead to differential changes in glucose metabolism. Along these lines, the similarities and differences of the metabolic impact of AP *versus* NTS *versus* hypothalamic glucose sensing remains an important future research direction. Given that hypothalamic glucose sensing also regulates feeding and triglyceride-enriched lipoprotein secretion (22, 25), the role of glucose sensing in the AP and NTS in feeding and lipid regulation remains to be clarified.

Of note, AP and NTS tissues contain both endothelial and glial isoforms of GLUT1 as reported by histochemical studies (26). We herein performed intraparenchymal infusion for both glucose and lentivirus expressing GLUT1shRNA that bypasses the blood-brain barrier, indicating that cellular (*i.e.*, glial, neuronal, and/or endothelial) GLUT1-mediated glucose uptake induced by glucose infusion is likely altered. It is to be acknowledged that our tools did not allow us to assess the relative contribution of GLUT1 in these specific cell types in the AP and NTS and future studies are warranted to assess the cell-specific contribution of GLUT1.

In summary, we discovered GLUT1- and pyruvate kinase–dependent glucose-sensing mechanism in the AP as well as the NTS of male rats regulates glucose production *in vivo*. HF feeding disrupts glucose-sensing mechanism downstream of pyruvate metabolism in the AP as well as the NTS. These findings highlight the role of AP and NTS in mediating glucose to regulate hepatic glucose production and identify potential HF-acquired molecular defect(s) of glucose sensing in the AP and NTS.

## Experimental procedures

### Animal preparation

Male Sprague Dawley rats weighing 260 to 280 g from Charles River Laboratories were used. Rats were housed individually and subjected to a standard 12-h light-dark (6:30 light, 18:30 dark) cycle. Rats had ad libitum access to drinking water and standard chow diet (Teklad Diet 7012, Envigo - 17% fat, 25% protein, and 58% carbohydrate; 3.1 kcal/g total metabolizable energy). A separate cohort of rats were switched to a 10% lard-enriched diet for 3 days before undergoing experiment (HF diet: TestDiet 57IR, Purina Mills, containing 34% fat, 22% protein, and 44% carbohydrate; 3.9 kcal/g total metabolizable energy). Rats were randomly assigned to HF groups, and those that did not develop hyperphagia were excluded from experimentation. All animal protocols were approved by the UHN Animal Care and Use Committee in accordance with the Canadian Council on Animal Care guidelines.

### Surgical procedures

Rats were anaesthetized with intraperitoneal injection of ketamine (60 mg/kg) and xylazine (8 mg/kg) for brain cannulation surgery. For AP-targeted cannulation surgeries, a single, 26-gauge, stainless steel guide cannula (C315G, Plastics One Inc) was stereotaxically implanted (0.0 mm lateral to the midline, 7.9 mm below cranial surface, 0.3 mm posterior the occipital crest). For NTS-targeted cannulation surgeries, a bilateral, 26-gauge, stainless steel guide cannula (C235G, Plastics One Inc) was stereotaxically implanted into the NTS (0.4 mm lateral to midline, 7.9 mm below cranial surface, at occipital crest). Seven days after the brain cannulation surgery, animals are anaesthetized (ketamine, 80 mg/kg; xylazine, 10 mg/kg) for vascular surgery, where indwelling catheters were implanted into the left carotid artery and right jugular vein for blood sampling and infusions. Postsurgical bodyweight and food intake were monitored, and animals were randomly assigned to the HF groups 3 days prior to experiment day to go on HF diet. Five days following the vascular surgery on experimentation day, rats were excluded if a minimum of 90% of their prevascular surgery bodyweight was not attained. Rats were randomly assigned into groups before the experiment.

#### Lentiviral infection

For AP or NTS lentiviral infections, after the brain cannulation surgery and while the rats were still anaesthetized, lentivirus expressing shGLUT1 (1.0 × 10^6^ infectious units; sc-270172-V, Santa Cruz Biotechnology Inc), shPK (1.0 × 10^6^ infectious units; sc-62821-V), or shMM (1.0 × 10^6^ infectious units; sc-108080) were injected. For AP-specific lentivirus injection, 2 μl of lentivirus was injected *via* the AP cannula over 15 min. For NTS-specific lentivirus injection, 3 μl of lentivirus was injected *via* the NTS bilateral cannulae over 10 min. The virus injection cannula was left in place for an additional 5 min after injection was completed to prevent backflow of the lentivirus. A dummy cannula and dust cap was inserted and secured afterward.

### Pancreatic (basal insulin)-euglycemic clamps

Rats were restricted to ∼80% of their previous food intake based on the 2 days immediately prior to achieving a ∼4 to 6 h fast before the clamp experiments and comparable postabsorptive nutritional status. Blood samples were first collected in conscious, unrestrained rats prior to receiving brain infusions. 0.9% saline, glucose (Sigma-Aldrich; 2 mM), pyruvate (Sigma-Aldrich; 5 mM), or dichloroacetate (Sigma-Aldrich; 1 mM) were infused at 0.33 μl/h throughout the clamps beginning at *t* = 0 min to *t* = 210 min. Concentration of glucose infused was chosen based on previous studies demonstrating an increase in dorsal vagal complex (DVC) and hypothalamic glucose concentration and activation of NTS and hypothalamic glucose mechanism (11, 19). For pyruvate infusion concentration, previous studies employing 5 mM for lactate infusion activated hypothalamic-sensing mechanisms to suppress glucose production (19). Considering equal conversion between pyruvate and lactate and approximating that every glucose molecule yields two pyruvate molecules, 5 mM was selected for pyruvate. Dichloroacetate concentration was selected based on a previous study demonstrating that at this concentration, hypothalamic glucose sensing is mimicked and glucose production is suppressed (11). Clamp methodology was performed as follows and as described. At *t* = 0 min, AP or NTS infusions as well as a primed, continuous infusion (PHD2000 syringe pump, Harvard Apparatus) of [3-3H]-glucose (PerkinElmer; 40 μCi bolus + 0.4 μCi infusion) was commenced and maintained until the end of the clamp experiment at *t* = 210 min to measure glucose kinetics using tracer-dilution methodology. Glucose turnover was calculated using the steady-state formulae where the rate of glucose appearance in blood was calculated using [3-3H]-glucose (27). Briefly, in the basal state, the total rate of tritiated glucose appearance corresponds to the endogenous glucose production at steady state. During the pancreatic-euglycemic clamps, endogenous glucose production was calculated by subtracting the exogenous glucose infusion rate from the rate of glucose appearance (*i.e.*, Glucose appearance = glucose production + exogenous glucose infusion rate) at steady state. Glucose disappearance corresponds to the rate of glucose appearance in steady state and is the tissue glucose uptake (or disposal or utilization) during the pancreatic-euglycemic clamps [*i.e.*, Glucose uptake (glucose disappearance) = glucose production + exogenous glucose infusion rate (glucose appearance)]. The pancreatic (basal insulin)-euglycemic clamp was initiated at *t* = 90 min with primed and continuous infusion (1.5 ml/h) of insulin (0.8 mU/kg of bodyweight/min), somatostatin (3 μg/kg/min), and a variable infusion of 25% glucose to maintain blood glucose at euglycemic level until *t* = 210 min. Basal period steady state calculation is based on *t* = 60 to 90 min and *t* = 180 to 210 min for clamp period steady state. Plasma samples were collected every 10 min into heparinized tubes and centrifuged for 30 s for the determination of [3-3H]-glucose–specific activity, plasma glucose levels, and C-peptide levels (Alpco, rat ELISA c-peptide kit). Plasma glucose levels were determined by glucose oxidase method using a GM9 glucose analyzer (Analox Instruments). To prevent hypovolemia and anemia, packed red blood cells were resuspended with equal volume to the extracted plasma of 0.2% heparinized saline and reinfused into the rat.

### Tissues collection

At the end of the clamps, rats were anesthetized, and 2 or 3 μl of bromophenol blue was infused over 20 or 30s into the AP cannula or bilateral NTS cannula, respectively, to verify correct placement of the cannula and aid in tissue collection. Rats that showed dye location beyond the correct AP or NTS location (Figs. S1*A* and 2*A*) were excluded from the studies. Animals are then immediately decapitated and the whole brain is isolated from the skull, bathed by PBS, and placed on a metal plate cooled by dry ice. Cerebellum is removed to allow a better view of the DVC region. Then, a ∼1.5 mm coronal section containing the DVC is obtained by cutting from the point of the obex to the end of the vagal triangle. Subsequently, a micro knife is used to isolate the AP and NTS tissue guided by rat brain atlas as well as with visual assistance from markers such as the cuneate nucleus and central canal. Tissues were immediately placed in liquid nitrogen and subsequently stored in −80 °C freezers for future analyses.

### Quantitative PCR analysis

AP (∼1 mg) and NTS (∼5 mg) tissue samples were weighed, and lysis buffer (20 μl/mg) was added accordingly. Tissue samples were then homogenized in lysis buffer for 3 min using the tissue homogenizer (Bullet Blender Storm Pro, Next Advance Inc). RNA was isolated using the PureLink RNA Mini Kit (12183025, Thermo Fisher Scientific), and RNA was quantified using Cytation 5 imaging reader (BioTek Instruments). Two micrograms of RNA then undergoes DNA digestion at room temperature for 10 min and this process is terminated by the addition of 25 mM EDTA and incubation at 70 °C for 15 min. Complementary DNA was generated using the SuperScript Vilo cDNA Synthesis Kit (11754050, Thermo Fisher Scientific). Quantitative PCR was performed using TaqMan Gene Expression master mix (4369016, Thermo Fisher Scientific) with qPCR machine (QuantStudio 7 Flex, Applied Biosystems). The primers used are as follows: rat ribosomal protein 18s (Rps18, Rn01428913_gH, Thermo Fisher Scientific), rat GLUT1 (Slc2a1, Rn01417099_m1), and mouse/rat pyruvate kinase (PKm, Rn00583975_m1). Relative gene expression was calculated using the ΔΔCt method in QuantStudio Real-Time PCR software 1.2, and samples were normalized to 18s rRNA as the reference gene.

### Statistical analysis

Statistical analyses were performed using GraphPad Prism. Unpaired Student’s *t* test was used in comparing two groups. One-way ANOVA with Tukey’s post hoc test was performed for groups of three or more with single variable. *p* < 0.05 was considered statistically different. All numerical results are presented as mean ± S.D.

## Data availability

All data presented are contained within the manuscript.

## Supporting information

This article contains supporting information.

## Conflict of interest

The authors declare no competing interests.
