# Supplementary material for: A glucose-sensing mechanism with glucose transporter 1 and pyruvate kinase in the area postrema regulates hepatic glucose production in rats
Source: J Biol Chem. 2023 Mar 23;299(5):104633. doi: 10.1016/j.jbc.2023.104633 (PMC10149203; doi:10.1016/j.jbc.2023.104633)
Supplement: Supporting Figures S1–S5 and Tables S1 and S2 [file mmc1.docx]

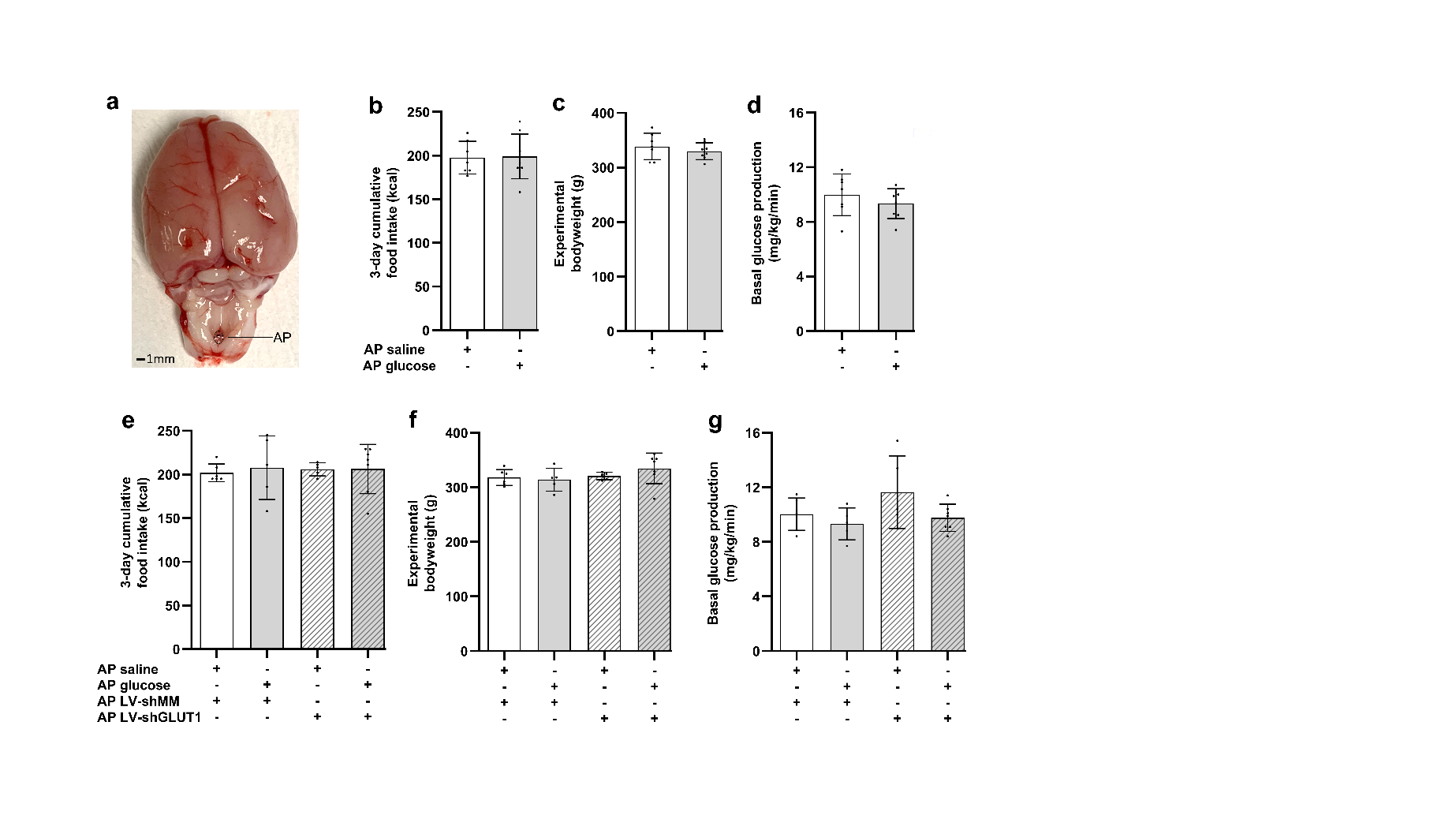


Supplementary Figure 1. *Extended data of Figure 1.* **a)** Image of rat brain collected post clamp experiments with bromophenol blue verifying area postrema (AP) cannula placement. **b)** 3-day cumulative food intake, **c)** bodyweight of rats prior to undergoing clamp experiments, and **d)** basal glucose production of rats receiving AP saline (n=7) or AP glucose (n=8). **e)** 3-day cumulative food intake, **f)** bodyweight of rats prior to undergoing clamp experiments, and **g)** basal glucose production of rats receiving AP saline with AP lentiviral infection of scrambled mismatch sequence (LV-shMM) (n=6), AP glucose with LV-shMM (n=5), AP saline with AP lentiviral infection of shRNA of GLUT1 (LV-shGLUT1) (n=5), or AP glucose with AP LV-shGLUT1 (n=7) . Data are shown as mean ± S.D.

**Supplemental Figure 1**


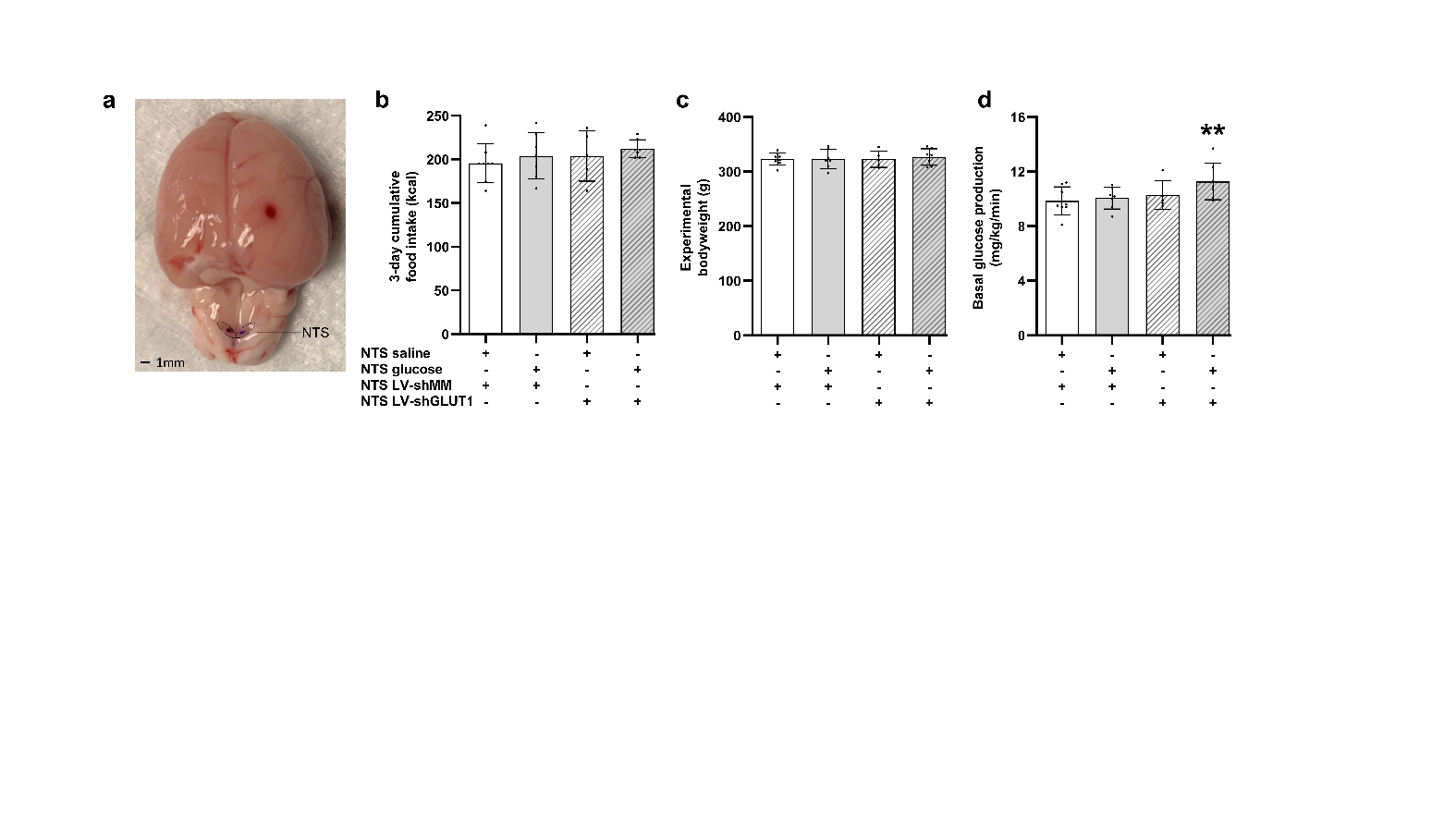


**Supplemental Figure 2**

Supplementary Figure 2. *Extended data of Figure 2.* **a)** Image of rat brain collected post clamp experiments with bromophenol blue verifying nucleus tractus solitarius (NTS) cannula placement. **b)** 3-day cumulative food intake, **c)** bodyweight of rats prior to undergoing clamp experiments, and **d)** basal glucose production of rats receiving NTS saline with dorsal vagal complex (DVC) LV-shMM (n=8), NTS glucose with DVC LV-shMM (n=7), NTS saline with DVC LV-shGLUT1 (n=5), or NTS glucose with DVC LV-shGLUT1 (n=7). ** p<0.01 vs other groups as determined by one way ANOVA followed by Tukey’s post hoc test. Data are shown as mean ± S.D.


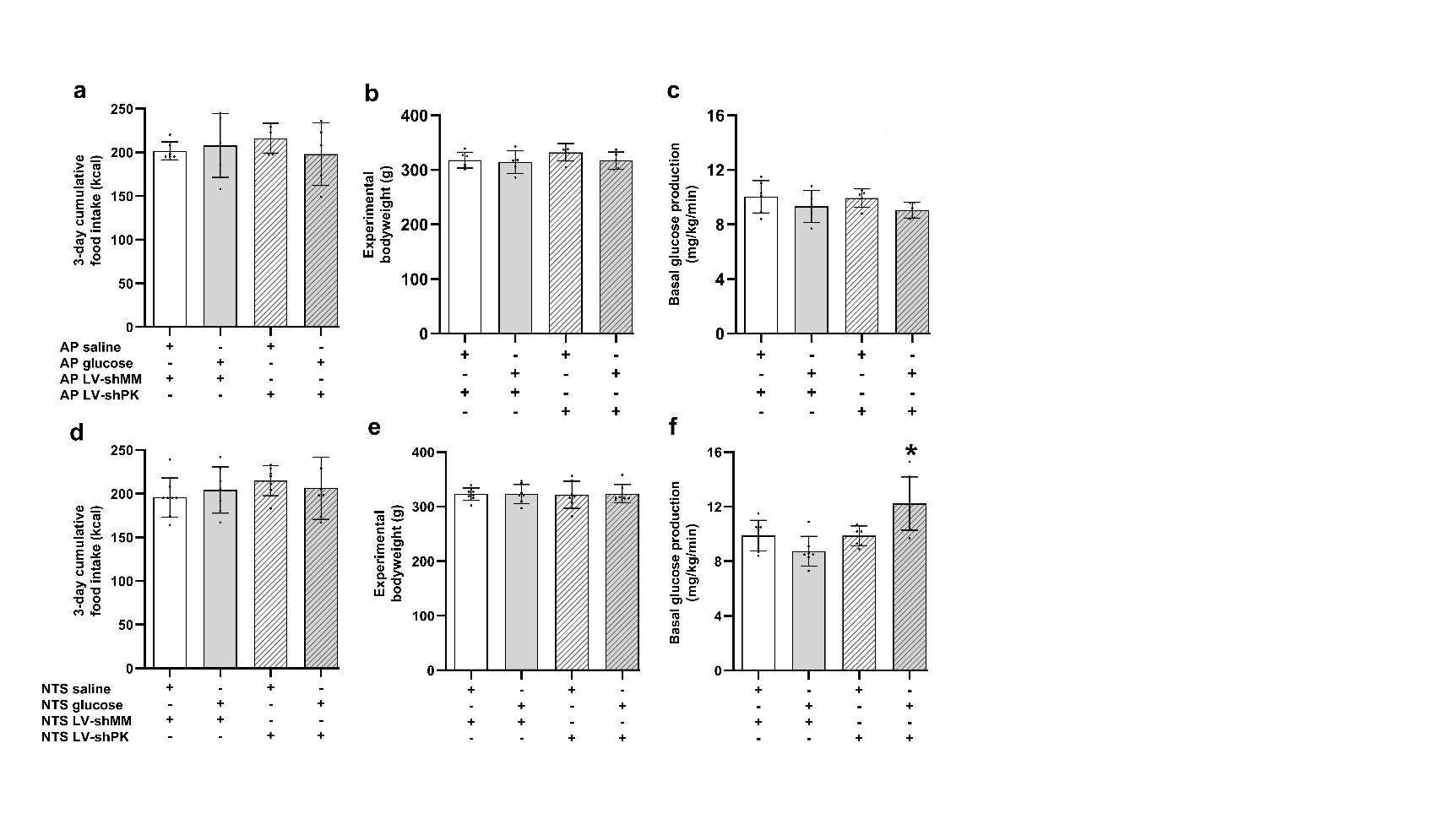


**Supplemental Figure 3**

Supplementary Figure 3. *Extended data of Figure 3.* **a)** 3-day cumulative food intake, **b)** bodyweight of rats prior to undergoing clamp experiments, and **c)** basal glucose production of rats receiving AP saline with AP LV-shMM (n=6), AP glucose with AP lentiviral infection of shRNA of pyruvate kinase (LV-shPK) (n=5), AP saline with AP LV-shPK (n=5), or AP glucose with LV-shPK (n=5). **d)** 3-day cumulative food intake, **e)** bodyweight of rats prior to undergoing clamp experiments, and **f)** basal glucose production of rats receiving NTS saline with DVC LV-shMM (n=8), NTS glucose with DVC LV-shMM (n=7), NTS saline with DVC LV-shPK (n=7), or NTS glucose with DVC LV-shPK (n=7). * p<0.01 vs other groups as determined by one way ANOVA followed by Tukey’s post hoc test. Data are shown as mean ± S.D.


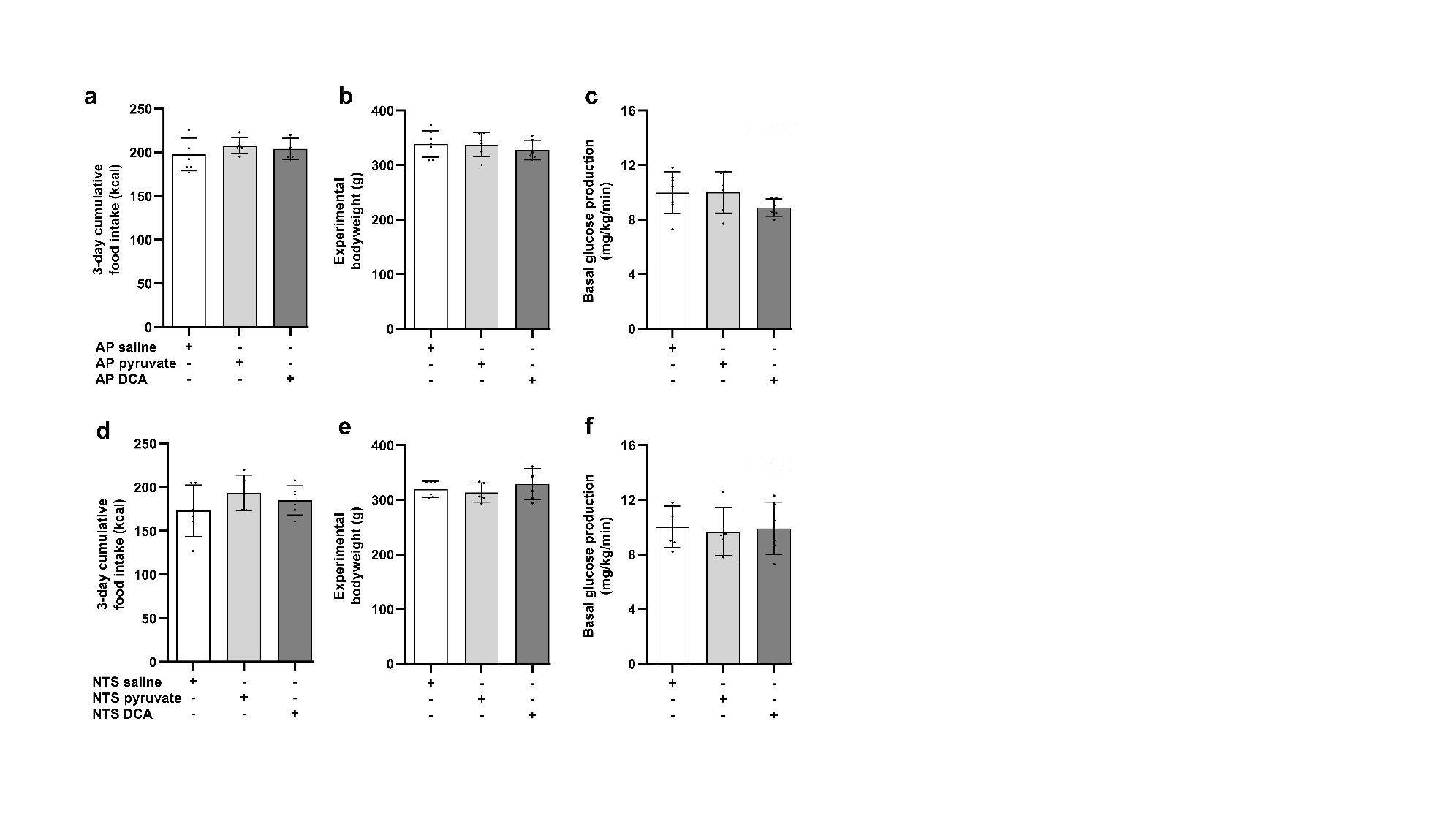


**Supplemental Figure 4**

Supplementary Figure 4. *Extended data of Figure 4.* **a)** 3-day cumulative food intake, **b)** bodyweight of rats prior to undergoing clamp experiments, and **c)** basal glucose production of rats receiving AP saline (n=7), AP pyruvate (n=6), or AP dichloroacetate (DCA) (n=6). **d)** 3-day cumulative food intake, **e)** bodyweight of rats prior to undergoing clamp experiments, and **f)** basal glucose production of rats receiving NTS saline (n=6), NTS pyruvate (n=5), or NTS DCA (n=6). Data are shown as mean ± S.D.


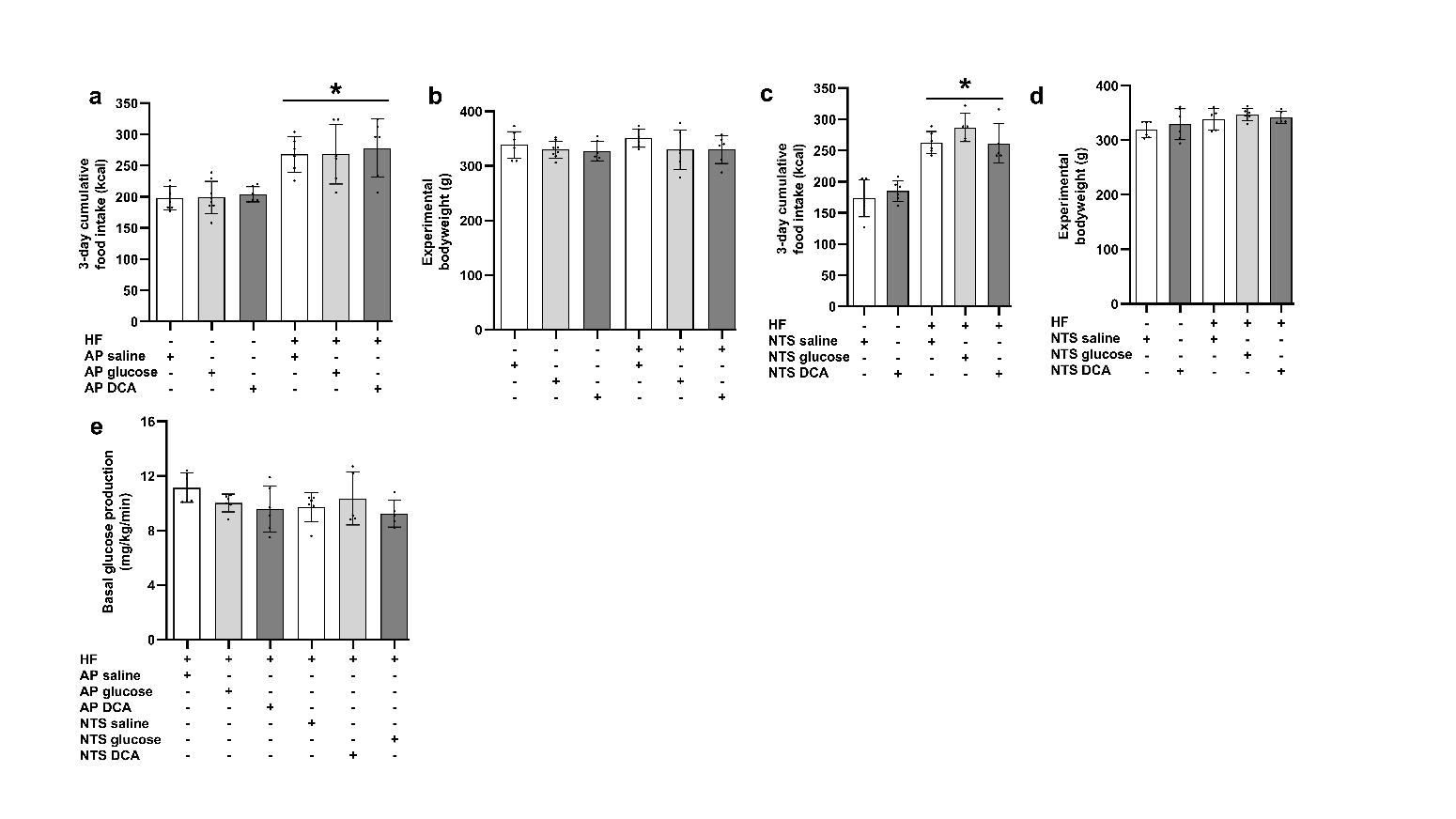


Supplementary Figure 5. *Extended data of Figure 5.* **a)** 3-day cumulative food intake, and **b)** bodyweight prior to undergoing experiment of rats that received AP saline (n=7), AP glucose (n=8), AP DCA (n=6), fed with high fat diet (HF) with AP saline (n=6), HF with AP glucose (n=6), or HF with AP DCA (n=6) during clamp experiment. **c)** 3-day cumulative food intake, and **d)** bodyweight prior to undergoing experiment of rats that received NTS saline (n=6), NTS DCA (n=6), HF with NTS saline (n=6), HF with NTS glucose (n=5) or HF with NTS DCA (n=5) during clamp experiment. **e)** Basal glucose production HF rats receiving AP saline, AP glucose, AP DCA, NTS saline, NTS glucose, or NTS DCA. *p<0.05 vs other groups as determined by one way ANOVA followed by Tukey’s post hoc test. Data are represented as mean ± S.D.

**Supplemental Figure 5**

**Supplementary Table 1** **Plasma glucose levels of rats during basal and clamp steady states of the pancreatic basal-insulin euglycemic clamps**

|  | **Basal**  **(60-90 min)** | **Clamp**  **(180-210min)** |
| --- | --- | --- |
| AP saline (n=7)  AP glucose (n=8)  AP saline + AP LV-shMM (n=6)  AP glucose + AP LV-shMM (n=5)  AP saline + AP LV-shGLUT1 (n=5)  AP glucose + AP LV-shGLUT1 (n=7)  AP saline + AP LV-shPK (n=5)  AP glucose + AP LV-shPK (n=5)  AP pyruvate (n=6)  AP DCA (n=6)  (HF) AP saline (n=6)  (HF) AP glucose (n=6)  (HF) AP DCA (n=6)  NTS saline + NTS LV-shMM (n=8)  NTS glucose + NTS LV-shMM (n=7)  NTS saline + NTS LV-shGLUT1 (n=5)  NTS glucose + NTS LV-shGLUT1 (n=7)  NTS saline + NTS LV-shPK (n=7)  NTS glucose + NTS LV-shPK (n=7)  NTS saline (n=6)  NTS pyruvate (n=5)  NTS DCA (n=6)  (HF) NTS saline (n=6)  (HF) NTS glucose (n=5)  (HF) NTS DCA (n=5) | 8.1 ± 0.2  7.5 ± 0.1  8.1 ± 0.2  7.9 ± 0.3  7.9 ± 0.2  7.9 ± 0.2  8.1 ± 0.2  7.8 ± 0.3  7.9 ± 0.2  7.5 ± 0.2  8.2 ± 0.4  8.3 ± 0.1  7.7 ± 0.1  8.0 ± 0.1  7.9 ± 0.2  8.0 ± 0.2  8.3 ± 0.2  7.6 ± 0.2  8.4 ± 0.2  8.0 ± 0.2  8.1 ± 0.4  7.5 ± 0.4  8.1 ± 0.3  7.8 ± 0.1  8.7 ± 0.3 | 7.5 ± 0.2  7.2 ± 0.1  7.3 ± 0.2  7.0 ± 0.2  7.5 ± 0.4  7.5 ± 0.2  7.6 ± 0.5  6.7 ± 0.3  7.1 ± 0.2  7.0 ± 0.3  7.8 ± 0.2  8.0 ± 0.2  7.6 ± 0.3  6.9 ± 0.2  7.5 ± 0.3  6.9 ± 0.6  7.2 ± 0.2  6.8 ± 0.2  7.2 ± 0.2  7.2 ± 0.2  7.0 ± 0.1  7.1 ± 0.4  7.9 ± 0.2  7.6 ± 0.1  8.5 ± 0.2 |

Data are presented as mean ± s.e.m.

**Supplementary Table 2 Plasma C-Peptide levels of rats at basal and clamp steady-states**

| (ng/ml) | **Basal**  **(90 min)** | **Clamp**  **(210 min)** |
| --- | --- | --- |
| AP saline (n=6, 5*)  AP glucose (n=6, 3*)  NTS saline (n=6, 4*)  NTS glucose (n=6, 5*) | 1.7 ± 0.21  1.5 ± 0.17  1.6 ± 0.30  1.4 ± 0.36 | 0.37 ± 0.10  0.25 ± 0.10  0.20 ± 0.06  0.24 ± 0.06 |

Data are presented as mean ± s.e.m. *Equal sample size was assayed for basal

and clamp samples. However, clamp sample size vs basal was lower for each

group because some values fell below the detectable range.
